# Incidence and Causes of Iatrogenic Hypoglycemia in the Emergency Department

**DOI:** 10.5811/westjem.2019.7.42996

**Published:** 2019-08-27

**Authors:** Chaitanya Chittineni, Brian E. Driver, Matthew Halverson, Jon B. Cole, Matthew E. Prekker, Vidhu Pandey, Tarissa Lai, Justin Harrington, Sean Zhao, Lauren R. Klein

**Affiliations:** * Hennepin County Medical Center, Department of Emergency Medicine, Minneapolis, Minnesota; † University of Minnesota School of Medicine, Department of Emergency Medicine, Minneapolis, Minnesota; ‡ Mercy Medical Center-North Iowa, Department of Emergency Medicine, Mason City, Iowa; § Aventura Hospital and Medical Center, Department of Emergency Medicine, Miami, Florida

## Abstract

**Introduction:**

Hypoglycemia is frequently encountered in the emergency department (ED) and has potential for serious morbidity. The incidence and causes of iatrogenic hypoglycemia are not known. We aim to describe how often the cause of ED hypoglycemia is iatrogenic and to identify its specific causes.

**Methods:**

We included adult patients with a chief complaint or ED diagnosis of hypoglycemia, or an ED glucose value of ≤70 milligrams per deciliter (mg/dL) between 2009–2014. Two independent abstractors each reviewed charts of patients with an initial glucose ≤ 50 mg/dL, or initial glucose ≥ 70 mg/dL with a subsequent glucose ≤ 50 mg/dL, to determine if the hypoglycemia was caused by iatrogenesis. The data analysis was descriptive.

**Results:**

We reviewed the charts of 591 patients meeting inclusion criteria. Of these 591 patients, 99 (17%; 95% confidence interval, 14–20%) were classified as iatrogenic. Of these 99 patients, 61 (61%) cases of hypoglycemia were caused by insulin administration and 38 (38%) were caused by unrecognized malnutrition. Of the 61 patients with iatrogenic hypoglycemia after ED insulin administration, 45 and 15 patients received insulin for hyperkalemia and uncomplicated hyperglycemia, respectively. One patient received insulin for diabetic ketoacidosis.

**Conclusion:**

In ED patients with hypoglycemia, iatrogenic causes are relatively common. The most frequent cause was insulin administration for hyperkalemia and uncomplicated hyperglycemia. Additionally, patients at risk of hypoglycemia in the absence of insulin, including those with alcohol intoxication or poor nutritional status, should be monitored closely in the ED.

## INTRODUCTION

Hypoglycemia is a serious and common condition that can cause seizures, loss of consciousness, and death. Emergency departments (EDs) often treat this pathology.^1^ A longitudinal study demonstrated that EDs treat more than 95,000 patients for hypoglycemia annually, comprising 3.4% of the entire diabetic population, of which 25% required hospital admission.^1^,^2^ While hypoglycemia is commonly caused by factors such as missing meals, wrong insulin medication or dose at home, hypoglycemia can also be caused by iatrogenesis.^1^,^3^

Iatrogenic hypoglycemia places the patient at risk of serious harm. This topic has been studied in hospitalized patients, but ED literature is limited.^1^,^3^ Iatrogenic hypoglycemia is often avoidable, and medical errors are common. In a voluntary survey of physician errors 76% of mistakes occurred during the initial testing and clinical assessment of the patient.^4^ ED patients may be at higher risk for hypoglycemia than the general population, especially those who receive insulin in the ED (for hyperkalemia or hyperglycemia, among other indications) and those who are acutely or chronically malnourished. Understanding the causes of iatrogenic hypoglycemia may assist emergency physicians in preventing this complication from occurring. In this study, we sought to determine the frequency and causes of iatrogenic hypoglycemia in an urban ED.

## METHODS

### Study Design and Setting

This was a retrospective, observational study conducted in the ED of an urban level 1 trauma center that cares for approximately 100,000 patients annually. The institutional review board approved this study.

### Selection of Participants

A data analyst identified adult (>18 years old) ED patients with hypoglycemia between 2009–2014 in the electronic health record (EHR) by searching for patients with an ED chief complaint or discharge diagnosis of hypoglycemia, or any ED glucose value ≤70 milligrams per deciliter (mg/dL) (local laboratory cutoff). In seeking to identify potential iatrogenic causes of hypoglycemia, we performed structured reviews of charts of patients with one or more initial ED glucose values of ≤50 mg/dL, and those with an initial glucose ≥70 mg/dL with one or more subsequent glucose values ≤50 mg/dL. We chose a cutoff of 50 mg/dL rather than a laboratory cutoff of 70 md/dL because glucose values ≤50 mg/dL have greater clinical significance and are more likely to be associated with patient harm; we selected a decrement from ≥70 mg/dL to ≤50 mg/dL because decrements of less than 20 mg/dL were less likely to be due to iatrogenic causes.

### Methods of Measurement

Patient demographics, chief complaint, ED diagnosis, and glucose values were abstracted from the EHR (Epic Systems, Verona, WI). Two trained abstractors independently performed a structured chart review for each identified patient.^[5]^ To determine whether the hypoglycemia was iatrogenic, the abstractors reviewed nursing and physician notes, laboratory results, vital signs, ED orders, and medications administered during the ED encounter. Iatrogenic hypoglycemia was defined as hypoglycemia (glucose ≤50 mg/dL) that occurred in the ED caused by 1) ED insulin administration, or 2) unrecognized or inadequately treated malnutrition. There are other causes of hypoglycemia (eg, sulfonylurea overdose, liver disease, and sepsis), but for the purposes of this study we only examined for the two most common causes of iatrogenic hypoglycemia.

**Population Health Research Capsule**

What do we already know about this issue?

*Iatrogenic hypoglycemia frequently occurs in the ED and may cause serious morbidity and mortality*.

What was the research question?

*How often does iatrogenic hypoglycemia occur in the ED and what are its causes*?

What was the major finding of the study?

*Patients receiving insulin for hyperglycemia or hyperkalemia or who have alcohol intoxication are at increased risk*.

How does this improve population health?

*Being aware of these high-risk populations may help ED physicians prevent future cases of iatrogenic hyperglycemia*.

Malnutrition was defined as any of the following: poor or reduced oral intake documented in the physician’s note; acute alcohol intoxication; chronic alcohol dependence; or inability to eat or drink in the ED (eg, an agitated patient who was sedated and placed in restraints, or patients with a nil per os diet order). We recorded the indication for insulin use if hypoglycemia was related to ED insulin administration. If the two reviewers disagreed whether the hypoglycemia was iatrogenic, a third abstractor reviewed the chart to make a final determination. To estimate interobserver agreement, we calculated an unadjusted kappa value for the initial two reviewers.

### Data Analysis

All data analyses are descriptive. Baseline characteristics are described using medians or proportions as appropriate. The proportion of ED visits with hypoglycemia deemed iatrogenic was reported, along with 95% confidence intervals (CI) and etiologies of iatrogenic hypoglycemia. Because there is no prior ED data, no a priori sample size was calculated. We used Stata (Version 12, Stata Corporation, College Station, TX) for all data analyses.

## RESULTS

Between 2009–2014, there were 2,858 patients who met initial inclusion criteria based on the chief complaint or ED diagnosis of hypoglycemia, or an ED glucose value ≤70 mg/dL. Of these 2,858 patients, we reviewed the charts of 591 (21%) who had an initial glucose ≤50 mg/dL or a decrement in glucose from ≥70 mg/dL to ≤50 mg/dL to determine if the hypoglycemia was iatrogenic. Baseline characteristics are presented in Table 1. Of these 591, 99 (17%; 95% CI, 14–20%) patients were determined to have iatrogenic hypoglycemia (Table 2). Interobserver agreement for iatrogenic hypoglycemia was 90% (kappa 0.63); disagreements were resolved by a third physician. The final rate reported reflects the outcomes of the adjudicated cases by the third reviewer.

The most frequent cause of iatrogenic hypoglycemia was insulin administration, for both uncomplicated hyperglycemia and for hyperkalemia. Details on the causes of iatrogenic hypoglycemia are presented in Table 2. Of those with iatrogenic hypoglycemia 40 patients (40%) had diabetes, while 59 (60%) did not.

## DISCUSSION

This study demonstrates that hypoglycemia in ED patients is commonly caused by iatrogenesis. In particular, insulin administration for hyperkalemia and uncomplicated hyperglycemia were frequent culprits. Unrecognized malnutrition in our population, especially in the context of alcohol intoxication, was another important cause of hypoglycemia that could have been prevented by more careful care.

Prior literature supports insulin administration as an important cause of iatrogenic hypoglycemia.^6^,^7^ Hyperkalemia is most prevalent in patients with end stage renal disease on chronic dialysis, and insulin is often administered in the ED to patients with hyperkalemia to shift potassium to the intracellular space until dialysis is available.^4^,^7^,^8^ Renal insufficiency leads to decreased insulin clearance, which increases the risk of hypoglycemia.^9^ A recent study demonstrated that 17% of ED patients who receive insulin for hyperkalemia develop hypoglycemia within three hours.^10^ The risk of hypoglycemia may be mitigated by administering smaller doses of insulin, larger doses of dextrose, or by more careful monitoring after insulin administration.^10^ With close monitoring even massive doses of insulin can be administered safely, as they are used to treat calcium-channel blocker and beta-blocker poisoning.^11^ Additionally, iatrogenic hypoglycemia is known to occur after ED insulin therapy for uncomplicated hyperglycemia; ED glucose reduction for uncomplicated hyperglycemia may lack value and consumes time and resources.^12^,^13^

Malnutrition, caused by alcohol intoxication or dependence, or reduced oral intake, was also found to be a common cause of iatrogenic hypoglycemia. Patients who present with chronic alcohol dependence or acute alcohol intoxication are likely to have depleted glycogen stores with concomitant gluconeogenesis inhibition secondary to poor nutrition and relative thiamine deficiency, and consequently are at higher risk for hypoglycemia.^14^ This patient population has many comorbidities; caring for acute intoxication and concomitant illnesses may distract from routine glucose monitoring, which is especially important when oral intake is limited by intoxication or parenteral sedation.^15^ Hypoglycemia in this patient population was recently found to be an independent predictor of subsequent critical illness.^16^ Additionally, these data support more careful monitoring of non-intoxicated patients who are placed nil per os.

We did not measure more patient-centered outcomes such as cost, hospital length of stay, encephalopathy from hypoglycemia, or mortality. However, preventing hypoglycemia is important for patient-safety, and hypoglycemia has been associated with poor outcomes.^17^–^19^ These data demonstrate and remind emergency physicians that care must be taken when administering insulin or caring for patients at risk of malnutrition.

## LIMITATIONS

We decided to search for only those with significant iatrogenic hypoglycemia (ie, those with nadir glucose ≤50 mg/dL who started at 70 mg/dL or higher), which may underestimate the true incidence of iatrogenic hypoglycemia. Additionally, malnutrition, though defined a priori, could have been interpreted subjectively due to potentially incomplete medical records and variations among abstractors. We attempted to mitigate this by having two abstractors review each patient chart. These limitations emphasize that the estimated value for iatrogenic hypoglycemia should not be viewed as a precise rate. Rather, it highlights the relatively common nature of this problem in the ED.

We care for a large socioeconomically disadvantaged population with a high burden of chronic disease, alcohol and drug dependence, and homelessness. These results may not generalize to other institutions that care for different patient populations. Our limited study population (n=99) also restrains the generalizability of these results. A larger multicenter study would provide greater external validity.

## CONCLUSION

In this retrospective study of hypoglycemia in the ED, patients without diabetes developed iatrogenic hypoglycemia more commonly than patients with diabetes. Insulin administration, especially in the context of hyperkalemia and uncomplicated hyperglycemia, was the most common cause of iatrogenic hypoglycemia. Additionally, patients at risk of hypoglycemia in the absence of insulin, including those with acute alcohol intoxication or poor nutritional status, must be vigilantly monitored while in the ED.

## Figures and Tables

**Table 1:** Baseline characteristics of patients with hypoglycemia.

Characteristic	All patients (N=591)	Patients with iatrogenic hypoglycemia (N=99)
Age, median (IQR) - years	51 (39–62)	49 (34–58)
Male gender - number (%)	346 (59)	63 (64)
Chief complaint - number (%)*		
Low blood sugar	134 (23)	0
Altered mental status	84 (15)	25 (25)
Abdominal pain	33 (6)	11 (11)
Chest pain	28 (5)	2 (2)
Shortness of breath	24 (4)	6 (6)
Fall	15 (3)	2 (2)
Dizziness	11 (2)	0
Weakness	11 (2)	3 (3)
Vomiting	10 (2)	1 (1)
High blood sugar	8 (1)	6 (6)
First recorded glucose, median (IQR) -mg/dL	48 (40–95)	97 (77–148)
Lowest recorded glucose, median (IQR) - mg/dL	41 (33–46)	42 (32–47)
Highest recorded glucose, median (IQR) - mg/dL	163 (116–238)	177 (121–237)

This table displays baseline characteristics for all patients meeting our initial inclusion criteria for hypoglycemia as well as patients deemed to have iatrogenic hypoglycemia.

* Only the 10 most common chief complaints are displayed in this table. Altered mental status is commonly used in our ED when a patient presents with alcohol intoxication.

*IQR*, interquartile range; *mg/dL*, milligrams per deciliter

**Table 2:** Study outcomes and emergency department management of hypoglycemia.

Parameter	Value (n=591)
Iatrogenic hypoglycemia number (%; 95% CI)	99 (17; 14–20)
Cause of iatrogenic hypoglycemia number (%)	
Insulin administered	61/99 (61)
Uncomplicated hyperglycemia	15/61 (25)
Diabetic ketoacidosis	1/61 (2)
Hyperkalemia	45/61 (74)
Malnutrition not recognized	38/99 (31)
Alcohol intoxication or dependence	29/38 (76)
Inability to eat in the ED	9/38 (24)
Parenteral ED management of hypoglycemia number (%)	
Dextrose containing fluids, 5% or 10%	54 (9)
Dextrose 50%	351 (59)
Glucagon	3 (1)

*ED*, emergency department; *CI*, confidence interval.
